# Proton pump inhibitors alter the composition of the gut microbiota

**DOI:** 10.1136/gutjnl-2015-310861

**Published:** 2015-12-30

**Authors:** Matthew A Jackson, Julia K Goodrich, Maria-Emanuela Maxan, Daniel E Freedberg, Julian A Abrams, Angela C Poole, Jessica L Sutter, Daphne Welter, Ruth E Ley, Jordana T Bell, Tim D Spector, Claire J Steves

**Affiliations:** 1 Department of Twin Research and Genetic Epidemiology, King's College London, London, UK; 2 Department of Molecular Biology and Genetics, Cornell University, Ithaca, New York, USA; 3 Department of Microbiology, Cornell University, Ithaca, New York, USA; 4 Clinical Age Research Unit, Kings College Hospital Foundation Trust, London, UK; 5 Division of Digestive and Liver Diseases, Department of Medicine, Columbia University Medical Center, New York, New York, USA

**Keywords:** PROTON PUMP INHIBITION, INTESTINAL MICROBIOLOGY, LACTIC ACID BACTERIA, LACTOBACILLUS, GASTRIC ACID

## Abstract

**Objective:**

Proton pump inhibitors (PPIs) are drugs used to suppress gastric acid production and treat GI disorders such as peptic ulcers and gastro-oesophageal reflux. They have been considered low risk, have been widely adopted, and are often over-prescribed. Recent studies have identified an increased risk of enteric and other infections with their use. Small studies have identified possible associations between PPI use and GI microbiota, but this has yet to be carried out on a large population-based cohort.

**Design:**

We investigated the association between PPI usage and the gut microbiome using 16S ribosomal RNA amplification from faecal samples of 1827 healthy twins, replicating results within unpublished data from an interventional study.

**Results:**

We identified a significantly lower abundance in gut commensals and lower microbial diversity in PPI users, with an associated significant increase in the abundance of oral and upper GI tract commensals. In particular, significant increases were observed in Streptococcaceae. These associations were replicated in an independent interventional study and in a paired analysis between 70 monozygotic twin pairs who were discordant for PPI use. We propose that the observed changes result from the removal of the low pH barrier between upper GI tract bacteria and the lower gut.

**Conclusions:**

Our findings describe a significant impact of PPIs on the gut microbiome and should caution over-use of PPIs, and warrant further investigation into the mechanisms and their clinical consequences.

**Significance of this study**

**What is already known on this subject?**

- Proton pump inhibitors (PPIs) are widely, and often over, used but recently have been associated with a number of side effects, including an increased risk of *Clostridium difficile* infection.
- The increased risk of infection may be mediated by alterations to the gut microbiota, as observed with antibiotics.
- Previous studies have demonstrated associations between PPI use and the gut microbiota, but have been limited in size.

**What are the new findings?**

- In a large healthy twin cohort, we identify significant associations between the composition of the gut microbiota and PPI use.
- The most striking association is an increase in Lactobacillales, particularly Streptococcaceae, in PPI users.
- The strongest associations replicated in a small interventional dataset indicating causality.
- Finally, we show that bacterial families increasing with PPI use are more likely to be pharyngeal, not gut, commensals.

**How might it impact on clinical practice in the foreseeable future?**

- The observed alterations to the gut microbiota with PPI use may be responsible for the observed increases in infection risk, and therefore provide targets for research to mitigate these risks.
- The potential consequences of these changes are motivation for caution against unnecessary provision of PPIs.

## Introduction

Proton pump inhibitors (PPIs) are used to increase gastric pH by suppressing acid production. They are pro-drugs, only becoming functional in the acidic environment of the stomach. Here, activated PPIs inhibit hydrogen–potassium pumps (H+/K+ ATPases), transmembrane proteins responsible for releasing hydrochloric acid into the lumen of the stomach. PPIs inhibit H+/K+ ATPases by binding covalently to the transmembrane domain, with return of acid production dependent on the turnover of new H+/K+ ATPases once PPIs have left the system.^1^

PPIs are frequently used to treat GI tract disorders such as bleeding peptic ulcers, erosive esophagitis, and gastroesophageal reflux.^2–4^ They are also used prophylactically to prevent stress ulcers and to reduce GI toxicity associated with certain medications, including non-steroidal anti-inflammatory drugs, aspirin, and steroids, sometimes despite a paucity of evidence.^5–8^ PPIs are one of the most profitable classes of drugs in the world^9^; however, the high cost to healthcare systems has led to investigations into possible over-utilisation. These show that over 70% of PPI prescriptions may be inappropriate,^10–12^ with the majority of over-utilisation stemming from unnecessary stress ulcer prophylaxis in patients who do not meet the evidence-based criteria, and a lack of re-assessment of PPI use in the community.^12^

The use of PPIs has generally been considered safe, with low reported incidences of serious adverse outcomes.^13–15^ However, recently a number of side effects have been identified, including nutritional deficiencies, increased risk of bone fracture, and risks of enteric and other infections^16–19^; notably, increased risks of community acquired pneumonia and *Clostridium difficile* infection where PPIs may carry a high risk equivalent to that of oral antibiotics.^20^ ^21^

The term microbiome refers to the ecology and functionality of the microbial population within an environment. Nearly every site of the human body has a distinct microbiome with bacterial composition determined by environmental and inter-microbial influences.^22^ ^23^ Using amplification and sequencing of the variable regions of the 16S ribosomal subunit it is possible to profile the taxonomic composition of the microbiome of a given sample.^23^ Application of this technique has shown changes to gut microbiota in a range of conditions, from IBD to obesity and frailty.^24–26^ Thus, factors affecting the microbiome have the potential to drive important secondary effects on health. For example, alterations to microbial communities caused by oral antibiotics may underlie their association with increased *C difficile* infection,^27^ and the same could be true for PPIs.

Previous small-scale case–control studies indicate that PPI use can influence the microbiome, but have been limited by focusing on younger individuals or patients presenting a GI disorder, with some conflicting results.^28–32^

Here we investigate the association between PPI usage and the gut microbiota in the largest study published to date, using 16S rRNA profiling of faecal samples collected from over 1800 healthy elderly twin volunteers, allowing adjustment for environmental and heritable factors influencing both PPI use and the microbiome. We identified significant alterations to the diversity and composition of the gut microbiota in PPI users, a number of which were replicated in an intervention study. We also identified a potential mechanism by which PPIs could induce such changes.

## Methods

### Microbiota composition analysis

One thousand and eighty-one faecal samples from the TwinsUK cohort had been sequenced as part of a previous study; a further ∼1000 twin samples were collected and processed under the same protocol producing reads with an average length of 253nt after barcode removal.^33^ Previously generated sequencing was combined with new data and quality filtering and ecological analysis performed using QIIME.^34^ Sequences were collapsed to operational taxonomic units (OTUs) using open reference clustering with Greengenes v13_8 at 97% sequence similarity. The OTU table was then sub-set to samples from twins with PPI usage data for use in subsequent analyses.

### Medication and GI indication data

PPI use was self-reported at multiple time points up to 10 years before and including microbiome assessment. Use was scored as positive if an individual had reported usage at any time, even if there was a more recent negative report. This method was chosen, as PPI use is often intermittent, the longevity of any PPI mediated microbiome effects are unknown, and most misclassifications would be non-users appearing as users, which would act to reduce the strength of any observed associations. Positive PPI use was recorded a median of 3 years before microbiota assessment (IQR 0.2–4.7 years).

GI indications were scored similarly based on self-reported or professionally diagnosed indications for PPI prescription. As for PPI use, multiple time points were available and individuals were considered positive if any indication had ever been reported. Positive GI indications were a median of 1.5 years from faecal sampling (IQR 0–3.8 years).

Self-reported antibiotic use within the previous month was recorded at the time of sample collection for the majority of individuals, with drug details provided where applicable. Binary scores created from these data were corrected to reflect reported treatments, removing individuals where the reported drug was not an antibiotic.

### Cohort and covariate data collection

Within TwinsUK 1827 individuals had both PPI data and faecal samples. The average age was 62 years (range 19–88 years) and 90% were female. The gender and age distribution resulted from historical study recruitment within the cohort.^35^ Physical measurements such as height and weight were measured at the time of sample collection.

Habitual dietary patterns were represented by the first five principle components (PCs) of food frequency questionnaires (FFQs) collected before sample collection. These have previously been shown to account for the majority of habitual diet variance and correspond to dietary types (given the names of fruit and vegetable rich, high alcohol, traditional English dieting and low meat diets, respectively).^36^ Frailty was quantified as a Frailty Index (FI) using the proportion of 39 binary health deficits that each individual displayed (see online supplementary table S1) from the Healthy Ageing Twin Study collected in 2007–2010.^35^ ^37^ Covariate distributions were analysed using two-tailed Wilcoxon rank sum tests to compare the distributions of PPI users and non-users, with a significance threshold of p<0.05.

### α Diversity

The 1827 samples had a mean OTU count of 82 130 (s=40 506, range 10 460–380 500). The OTU table was rarefied to a depth of 10 000 sequences and used to generate Shannon, Chao1 and phylogenetic diversity indices, as well as observed OTU counts. One-tailed Wilcoxon rank sum tests were performed to test for lower diversity in PPI users versus non-users,^32^ taking a significance threshold of p<0.05 on the full set of 1827 individuals.

Mixed effects models were created using the lme4 package in R,^38^ with α diversity metrics as the response variable to assess the ability of PPI status to predict diversity. Technical covariates included sequencing run and depth of sample sequencing. Other covariates included family, twin structure (a variable of unique values the same for monozygotic (MZ) but different for dizygotic (DZ) twins), diet (the first five PCs from FFQs), age, body mass index (BMI), FI (root normalised), and GI indication status. The Anova function was used to compare the ability of models with and without PPI status to predict each α diversity metric, using the subset of 1200 individuals with complete covariate data.

### OTU and taxonomic associations

Mixed effects models were again used to identify associations between PPI use and OTU and taxa abundances on 1200 individuals having complete covariate data. OTUs present in <25% of individuals were discarded and the remaining counts converted to log transformed relative abundances (with the addition of 10^−6^ for zero counts). OTU abundances were used as response variables with covariates as above also including the Shannon index, to reduce associations with OTU markers of α diversity. The ability of models including and not including PPI status as a covariate to predict abundance of each OTU was quantified using the Anova function in R. p Values were FDR (false discovery rate) corrected using the ‘qvalue’ package with a significance threshold of 5%.^39^ OTU counts were collapsed by shared taxonomy at all taxonomic levels. Modelling was carried out for each level individually in the same manner as for OTUs. These analyses were repeated within the subset of individuals who had not used antibiotics.

### Interventional study replication

To further assess the possible causal link between exposure to PPIs and the observed taxonomic changes in TwinsUK, we re-analysed data from a previously published crossover study. Methods for this study have been described.^31^ In brief, 12 healthy adult volunteers not exposed to antibiotics within the previous 12 months each took 40 mg of omeprazole twice daily for 4–8 weeks, and donated stool samples before and after the PPI course. Bacterial DNA was extracted from all samples and the V4 region of the 16S rRNA gene was amplified using a primer set identical to that used in the TwinsUK study. As for the TwinsUK cohort, the Greengenes database was used for final taxonomic assignments. To best compare data, we assessed the taxonomic changes within the samples from immediately before and immediately after 4 weeks of omeprazole. We analysed taxa that were significantly associated with PPI in TwinsUK and present in the majority of individual specimens (>50%) in the intervention study. We assessed the magnitude and directions of within-individual changes using rank-sum tests (when the distribution of data was not normal) or paired t tests. Taxonomies assigned as ‘Other’ against the Greengenes reference were not included as they were not comparable between sets.

## Results

### PPI use in the TwinsUK cohort

Intermittent data on self-reported PPI usage and GI health over a ∼10 year time span was available for 1827 individuals, comprising 374 DZ twin pairs, 410 MZ twin pairs and 259 singletons; 90% were female, with an average age of 62 years. Within this set, 892 (49%) had reported some form of GI indication for PPIs, 229 (12%) had been prescribed PPIs at some point (only 24 having used PPIs without any GI indication), and 704 (39%) had reported neither PPI prescription nor GI indication.

### PPI use is associated with age, BMI, frailty, and diet

**Figure 1:**
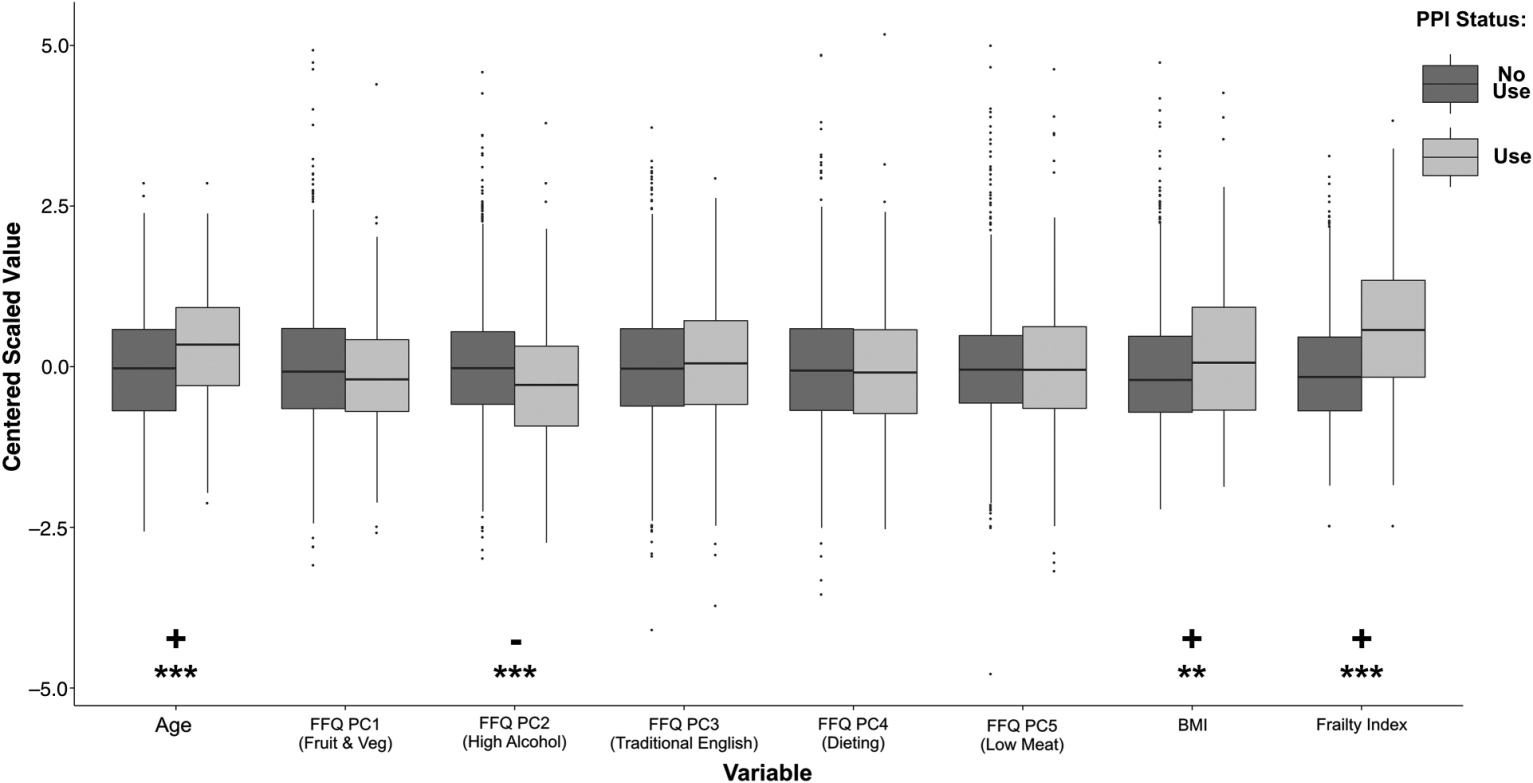
Distributions of covariates included for analysis, compared between proton pump inhibitor (PPI) users and non-users. Wilcoxon rank sum tests were carried out to compare the distribution of covariates in the modelling analysis. All variables were on a different scale so were centred and scaled before plotting. PPI users were older, frailer, had higher body mass index (BMI) and lower scores on the high alcohol food frequency questionnaire (FFQ) principle component (PC). Significant differences are indicated where ***p<0.001 and **p<0.01.

A number of covariates were selected. These included: age, diet as quantified using the first five PCs from FFQs, BMI, and frailty. The association of these with PPI use was assessed in the subset of individuals with complete covariate data (n=1200, 175 having use PPIs) (figure 1). PPI users were significantly older (p<10^−6^), frailer (p<10^−15^), and had higher BMI (p=0.002). They were also found to be significantly lower scoring on FFQ PC2 (p=0.0003), a dietary component related to high alcohol intake.^35^

### Significantly lower diversity in the gut microbiome of PPI users

**Figure 2:**
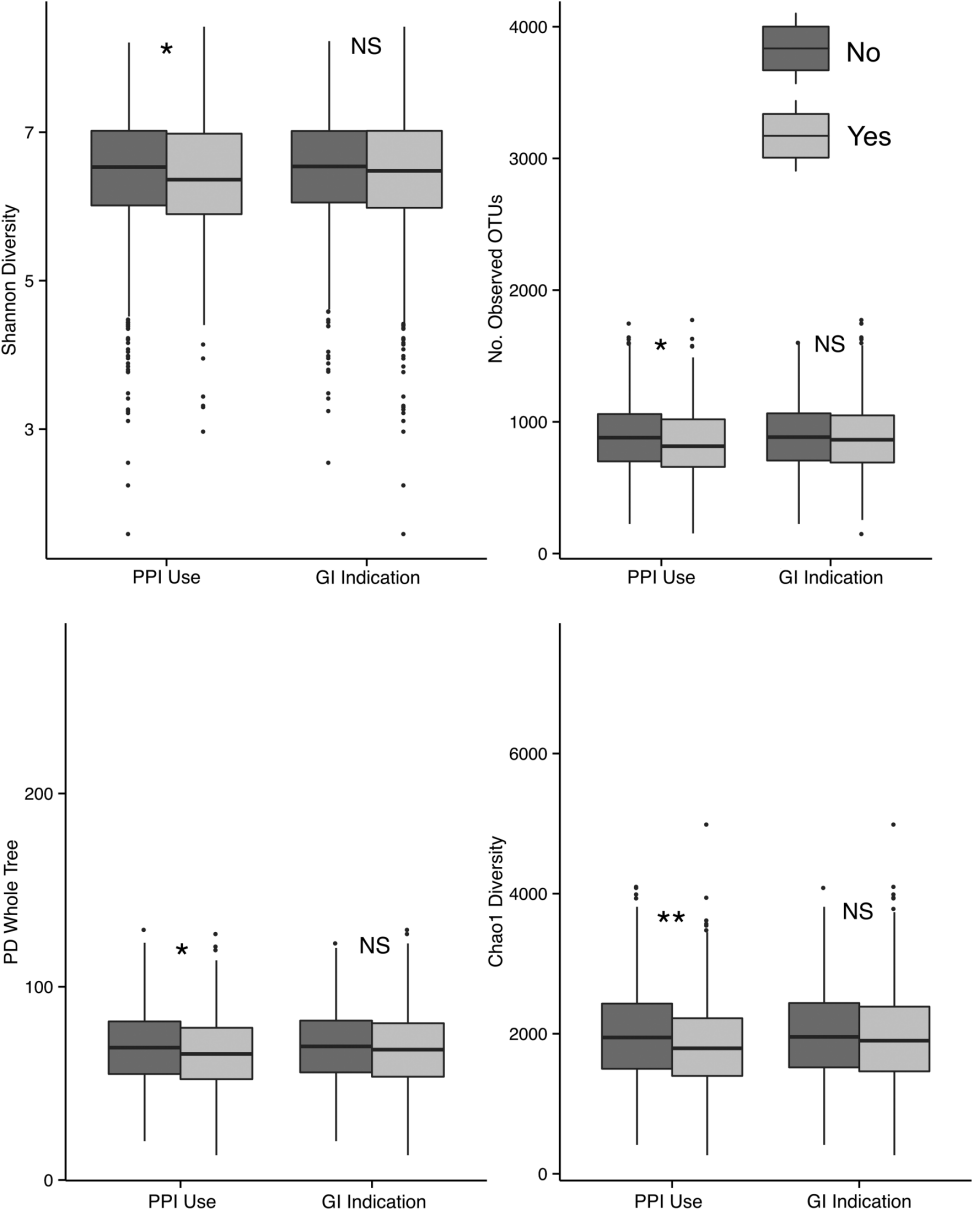
Comparison of α diversity in proton pump inhibitor (PPI) users and non-users, and in individuals with and without GI indications. Four metrics of α diversity were calculated on rarefied samples and one-tailed Wilcoxon rank sum tests carried out to test for significantly lower diversity with PPI use, or with GI indication. Significantly lower diversity was observed for all metrics in PPI users versus non-users (*); no difference was found splitting by GI indication (NS).

There was significantly lower (p<0.05) diversity in the gut microbiota of PPI users compared to those not using PPIs with all diversity indices (figure 2). There was no significant difference, with any diversity metric, between the individuals with GI indications compared to those without.

The observed negative association between PPI use and α diversity did not withstand adjustment for family and twin structure, BMI, age, frailty and GI indication, in the 1200 individuals with complete data (see online supplementary table S2).

### PPI use is associated with specific taxonomic abundances

Modelling of OTU abundances against PPI use identified 22 OTUs with significantly lower abundance in PPI users; all were assigned to the phylum Firmicutes. There were 32 OTUs positively associated with PPI use, 20 from the order Bacteroidales and seven assigned to the *Streptococcus* genus. The strongest association was with a *Bifidobacterium* OTU (q<10^−4^, β=0.45), followed by a *Streptococcus* assigned OTU (q<10^−4^, β=0.44) (see online supplementary table S3).

**Figure 3:**
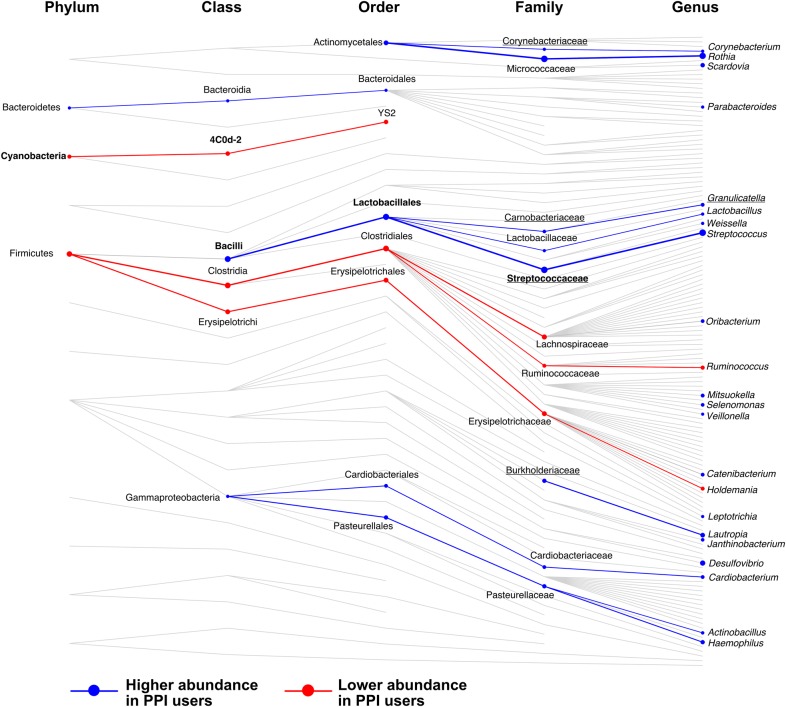
Summary of taxonomic associations with proton pump inhibitor (PPI) use. Shown are all collapsed groups used in taxonomic association analyses that had complete taxonomic assignment (not including collapsed species). Connecting lines highlight the taxonomic relationships between groups (not considering genetic relatedness). Taxa significantly associated with PPI use are highlighted with circles, larger circles representing a larger absolute coefficient of association. Association analyses were carried out at each taxonomic level independently. Taxa at higher abundance with PPI use are shown in blue and at lower abundance in red. Lines connecting taxa of similar association are also coloured and weighted by the average coefficient between the taxa. Names are shown for significant results only. Those in bold retained significance between 70 discordant monozygotic (MZ) twins, and underlined taxa replicated in analysis of interventional study data.

To identify specific taxonomic relationships, modelling was repeated against OTU abundances collapsed by shared taxonomic assignment at various depths of classification (see online supplementary table S4). A summary of significant associations is shown in figure 3.

Seven collapsed species were negatively associated with PPI use and were all assigned to Erysipelotrichales or Clostridiales (except from one Cyanobacteria). At the level of genera, nine were found to be negatively associated with PPI use, which were largely Firmicutes, with members of the family Erysipelotrichaceae being the most significantly decreased. Five families were negatively associated with PPI use, most strongly, Lachnospiraceae (q=0.004, β=−0.35) and Ruminococcaceae (q<0.0007, β=−0.26).

There were 24 species positively associated with PPI use. These belonged to the phyla Actinobacteria, Bacteroidetes, Firmicutes (particularly Lactobacillaceae and Clostridiales) and Proteobacteria. The largest increases observed with PPI use were the species *Rothia mucilaginosa* (q<10^−6^, β=0.51) and *Streptococcus anginosus* (q<10^−6^, β=0.48). We observed 24 genera that were positively associated with PPI use. The most significantly increased were *Rothia* (q<10^−5^, β=0.45) and *Streptococcus* (q<10^−6^, β=0.47). Ten families were significantly positively associated with PPI use, the most significant being Streptococcaceae (q<10^−6^, β=0.46) and Micrococcaceae (q<10^−5^, β=0.46).

### Taxonomic associations with PPI use are independent of antibiotic use

Self-reported oral antibiotic usage data were available for 1 month before faecal sample collection for 1039 of the 1827 individuals. Antibiotic use was significantly associated with PPI use within this set (χ^2^(1, N=1309)=8.88, p<0.002), where 16% of PPI users had used antibiotics compared to only 8% of individuals who had not used PPIs. To ensure this enrichment was not influencing the observed associations, modelling analyses were repeated within a subset of 705 individuals that had reported no antibiotic use and had complete covariate data (see online supplementary table S5).

At all levels of analysis, from OTU to phylum, results reflected those of the wider set. The number of significant associations was reduced because of the smaller sample size, but the majority of associations were retained, particularly the strongest positive associations with Streptococcaceae and other Lactobacillales, and the negative associations observed with the class Clostridia. These results show that the observed microbiome associations with PPI use are independent of increased antibiotic utilisation.

### Significant associations between discordant twin pairs

The influence of PPIs on the microbiota of 70 MZ twins discordant for PPI use was investigated to control for shared environmental and genetic effects (see online supplementary methods).

**Figure 4:**
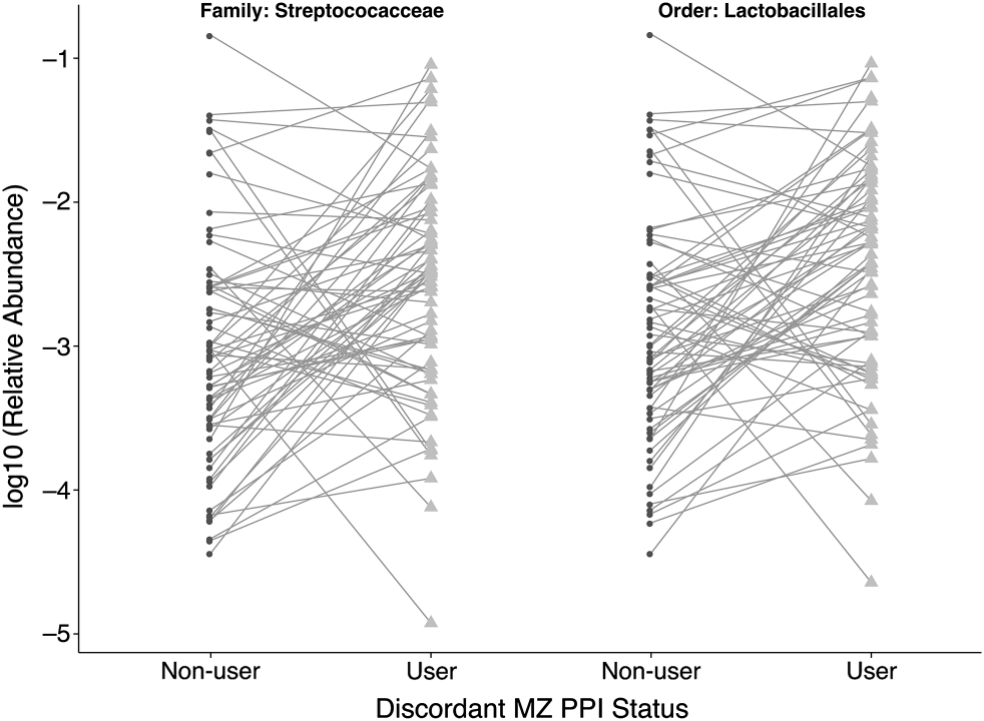
Paired plots of the relative abundances of Streptococcaceae and Lactobacillales within monozygotic (MZ) twins discordant for proton pump inhibitor (PPI) use. These were the only collapsed family and order traits found to be significantly different in discordant MZ twin pairs, both higher in abundance in PPI users.

No significant differences in the abundances of any OTU, species or genera were observed between discordant MZs. The Streptococcaceae family had a significantly higher abundance in PPI users within discordant twins (q=0.04, ×2.9 higher), as did its parent order Lactobacillales (q=0.02, ×2.6 higher) (figure 4).

At higher taxonomic levels, significant changes were observed between twins for the classes Actinobacteria (q=0.03, ×1.3 higher in PPI users), Bacilli (q=0.03, ×1.9 higher), and 4C0D-2 (q=0.03, ×0.3 lower), and the phyla Actinobacteria (q=0.04, ×1.1 higher), Cyanobacteria (q=0.04, ×0.33 lower), and Verrucomicrobia (q=0.04, ×0.4 lower).

### PPI microbiota associations replicate in an interventional study

From the 96 collapsed taxonomies significant in the TwinsUK set, 63 were found in at least 50% of the interventional set and considered for replication (see online supplementary table S4). Within these, seven were significantly associated with PPI use in the intervention, all increasing in abundance after 4 weeks of PPI use. These were unassigned species belonging to the genera *Streptococcus* and *Granulicatella*, the *Granulicatella* genus, and the families Carnobacteriaceae, Streptococcaceae, Burkholderiaceae, and Corynebacteriaceae. All belonged to the order Lactobacillales except Corynebacteriaceae and Burkholderiaceae, which are from the orders Actinomycetales and Burkholderiales. Within this size-limited intervention the strongest taxa associations, particularly Lactobacilli, appear to be driven by PPI use.

### PPI use associated with a higher abundance of pharyngeal bacteria in the gut

Body site of origin of the altered bacteria was investigated to shed light on the mechanism driving observed associations. Data from the human microbiome project (HMP) was used as a reference to determine body site preferences of bacterial families (see online supplementary methods and supplementary table S6). It is worth noting that individual species within each family may be commensals at different and multiple sites. Here we simply aimed to determine overall if families were more frequently identified at particular body sites.

Within the five families found to associate negatively with PPI use in TwinsUK, four were more common in the gut, with two also being found in abundance in the mouth/throat. The exception was Cyanobacteria, which was not biased towards any site in the HMP data. All 10 families positively associated with PPI use (including four replicated in the interventional study) displayed site preference. The six strongest, most significantly associated families were enriched in the mouth/throat with one also in abundance in the skin/nose, one was only enriched at the skin/nose sites, and two were most commonly found at vaginal sites. The only family most common to the gut and with increased abundance with PPI use was Burkholderiaceae. Overall, families with significantly reduced abundance with PPI use were more often found in the gut in the HMP data; while families with significantly higher abundance with PPI use were more often found in the mouth/throat, skin/nose or vaginal sites (likely a result of the large number of Lactobacillaceae commensals found here).^23^

To determine if this trend applied to all families, including those not significantly associated with PPI use, coefficients of association of each family with each site in the HMP data were correlated against families’ associations with PPI use in the TwinsUK data. There was a non-significant negative correlation between the association with PPI use and with the gut (ρ=−0.23, p=0.07), and a non-significant positive correlation with vaginal coefficients (ρ=0.2, p=0.12). However, significant positive correlations were observed between the association with PPI use and the association with the mouth/throat (ρ=0.38, p=0.0019) and the skin/nose (ρ=0.36, p=0.003) sites.

## Discussion

We have profiled the effects of PPI use on the gut microbiome in by far the largest study to date, and considered a number of possible confounders including host genetics. We have demonstrated that PPI use is associated with an altered composition of the gut microbiota, and a moderately lower diversity. In all three analyses, the large observational study, between discordant twins, and the interventional replication, PPI use was associated with increases in the Lactobacillales order, and in particular the family Streptococcaceae. Further, we show these effects could result from downward movement of upper tract commensals.

We observed a significant reduction in microbial diversity with PPIs. However, it was a small difference and became non-significant after adjusting for covariates. This may be due to confounding effects and/or reduced power of the smaller sample. Variables we found to associate with PPIs, such as BMI, frailty, and antibiotic use, are also known to reduce diversity.^24^ ^26^ ^40^ Therefore, it is likely that these factors are partly responsible for the observed lower diversity in PPI users; such confounders were also not accounted for in previous observations of decreased diversity with PPI use.^32^ This is further supported by a number of studies where no major changes in diversity have been observed.^28^ ^30^

There was a clear association between the composition of the microbiome and PPI utilisation. Collapsing by taxonomic assignment revealed the lineage specificity of these associations, in particular to those containing Streptococcaceae and other Lactobacilli. A number of these associations have been identified in smaller studies. For example, 8 weeks of PPI use was found to increase the abundance of Actinomycetales and Lactobacillales in the oesophagus of 34 patients suffering from heartburn.^29^ An increase in *Streptococcus* was also observed with PPI use in a case–control study of 116 children,^30^ and previous analyses of the intervention study data utilised within this study also identified similar increases in Streptococcaceae and Micrococcaceae.^31^ These studies also identified changes not present in our study, for example, increases in Gemellales, Enterococacceae, and Staphylocacceae.^29^ ^31^

We found families with higher abundance with PPI use to be frequent commensals of the oral, throat, nasal, and skin communities. We hypothesise that under normal circumstances gastric acid acts as a barrier to progression down the GI tract for pharyngeal commensals and environmental bacteria, which are not well adapted to low pH. Treatment with PPIs removes this barrier allowing colonisation by these bacteria further along the GI tract, eventually translating to the detected increased abundance in faecal samples. As observations are based on relative abundances, the observed lower abundance of gut commensals likely reflects the increase in other taxa, rather than a reduction in absolute levels.

PPIs may also act on specific bacterial taxa directly. Previous evidence suggests that they may have antimicrobial action against *Helicobacter pylori*.^41^ ^42^ Also, at least one *Streptococcus* species is known to have P-type ATPase transporters belonging to the same enzyme family as the human H+/K+ ATPase targeted by PPIs.^43^ Bacterial targets for direct PPI interactions could drive species-specific compositional changes.

There are limitations to the study. The TwinsUK data are observational, although our key findings were confirmed between twin pairs and replicated in data from a small prospective controlled trial. PPI use and GI indication were self-reported and over a wide timespan from faecal sampling. However, misclassification of exposures should only serve to reduce the strength of observed associations. We have also omitted duration of PPI use as accurate data were not available, which should be considered in future investigations as it may influence the strength of microbiome associations. However, the lack of duration data would tend to dilute our associations as short and long-term users are classified together. Similarly, we did not consider the effects of withdrawal of PPI treatment, which may be important given that dysbioses resulting from antibiotic use can have long lasting effects.^40^

Antibiotic use was not scored for all individuals within this study; we have also not considered the effects of particular classes of antibiotics, dosage and duration of courses, or antibiotic use before the previous month. However, the robustness of our observations within the subset of individuals who had not used antibiotics shows that they are independent of the effects of recent antibiotic exposure. This study was also limited to faecal sampling. While the observations in the gut are robust, they offer no insight into the distribution of these bacteria along the GI tract. Sampling of multiple sites combined with culture experiments would determine the distribution of living bacteria, and the influence of upper tract community composition on subsequent changes in the gut. In vitro studies will also be required to elucidate whether our observations are driven by pH changes, direct drug interactions, or a combination of both. This will be particularly important to determine if these effects occur with other classes of acid-suppressing medication.

The associations reported here are of clinical importance. *C difficile* affects nearly half a million people in the USA annually,^44^ and is known to capitalise on alterations to the normal gut microbiota.^27^ The increased risk of enteric infection with PPI use may similarly be mediated through changes to the GI microbiome. It has been shown that a high abundance of *Streptococcus* in the gut predisposes mice to *C difficile* colonisation, while *Lachnospiraceae* are protective.^45^ On this basis, we observed taxonomic changes that would be expected to promote *C difficile* infection. Further investigations into the microbiome-mediated determinants of *C difficile* infection will be important to understand how to mitigate the risk associated with PPI use.

A further consequence for consideration is the potential for the GI tract to become a reservoir for potential pathogens at alternate body sites. A significant increased risk of community-acquired pneumonia has been observed with PPIs (relative risk 1.98 for users vs ex-users),^18^ and has been observed specifically for *Streptococcus* derived pneumonia.^46^ There is speculative evidence of bacterial exchanges between the gastric and lung fluids,^30^ ^47^ and depletion of the gut microbiota reduces immune mediated resilience to pneumococcal pneumonia in mice.^48^ PPI use has also been shown to increase the risk of spontaneous bacterial peritonitis and overall bacterial infection in patients with cirrhosis and ascites,^19^ suggesting PPI use may pose a higher risk to individuals already susceptible to infection and other complications; for example, the elderly and the more frail or more obese individuals, whom our study indicates are more likely to be prescribed PPIs.

The described associations between PPI use and the gut microbiome warrant further research to better understand the driving mechanisms and their consequences, and are a further reason to reduce unnecessary prescribing.

## Supplementary Material

Web supplement 1

Web supplement 2

Web supplement 3

Web supplement 4

Web supplement 5

Web supplement 6

Web supplement 7

Web supplement 8

Web supplement 9
