# Supplementary material for: Proton pump inhibitors alter the composition of the gut microbiota
Source: Gut. 2015 Dec 30;65(5):749–56. doi: 10.1136/gutjnl-2015-310861 (PMC4853574; doi:10.1136/gutjnl-2015-310861)
Supplement: Web supplement 1 [file gutjnl-2015-310861-s1.pdf]

SUPPLEMENTARY METHODS

Twin pair analyses

70 pairs of MZ twins, within the 1827 individuals, were identified that were discordant for PPI use. Comparison of OTUs and collapsed taxonomies between the PPI users and non-users within pairs was carried out using two-tailed paired Wilcoxon signed rank tests on log transformed relative abundances. The resultant p-values were FDR corrected using the q-value package in R with a significance threshold of 5%. Abundances were not adjusted for any covariates in these analyses.

Site specificity of families in the Human Microbiome Project

Pre-processed 16S data was obtained from the Human Microbiome Project (HMP) in the form an OTU table.[1] This was collapsed at the family level using QIIME. Sample sites were renamed to broader categories of nose, mouth, throat, skin, vagina and gut (Supplementary Table S7). Principle components analysis was then carried out on all samples using log transformed relative abundances of families (Supplementary Figure S8). This showed at the family level there was broad overlap of throat and mouth, and skin and nose samples; therefore these groups were combined resulting in four sites for comparison.

Family abundances at each site were then compared using Kruskall-Wallis one-way ANOVA tests in R. Resultant p-values were Bonferroni adjusted and any families with $p < 0.05$ were considered to have significantly different distributions across sites. Ad hoc pair-wise comparisons were carried out on significant families using the Nemenyi test from the PMCMR package.[2] Where there were between site differences with $p < 0.05$, boxplots were used to manually assign site specificity of a family, assigning multiple sites where necessary.

Mixed effects models were made with family abundance as the response with sample site as a random effect using the coefficient of each family with each site as a measure of its site based association. To assess how the site based association of a family related to its association with PPI use, the coefficients of families at each site in the HMP data were correlated against the coefficient of families with PPI use in the TwinsUK data using Pearson correlations in R. This included 64 families that were found in both sets.

SUPPLEMENTARY LEGENDS

S1. Summary of the aspects covered by the health domains considered in the construction of the frailty index.

S2. Table of alpha diversity associations with PPI use after adjustment for covariates.

S3. Significant results from modelling OTU associations with PPI use

S4. Significant taxa associations with PPI use and replication results in the interventional data set.

S5. Significant results from mixed effects modelling of PPI and OTU and taxa associations in the subset of individuals with complete covariate data and no antibiotic use within the previous month.

S6. Table of HMP derived site specificities for collapsed families.

S7. Table showing HMP site definition mappings to the broader categories used in this study.

S8. Plot of HMP samples by PC1 and PC2 of PCA from relative abundances of all collapsed families. Samples are coloured by site. This clustering was used to group sites into the four categories: gut, mouth/throat, skin/nose and vagina.

SUPPLEMENTARY REFERENCES

1 The Human Microbiome Project Consortium. Structure, function and diversity of the healthy human microbiome. *Nature* 2012;**486**:207–14. doi:10.1038/nature11234

2 Pohlert T. PMCMR: Calculate Pairwise Multiple Comparisons of Mean Rank Sums. 2015.
