# Supplementary material for: Proton pump inhibitors alter the composition of the gut microbiota
Source: Gut. 2015 Dec 30;65(5):749–56. doi: 10.1136/gutjnl-2015-310861 (PMC4853574; doi:10.1136/gutjnl-2015-310861)

Supplementary S8. Plot of HMP samples by PC1 and PC2 of PCA from relative abundances of all collapsed families. Samples are coloured by site. This clustering was used to group sites into the four categories: gut, mouth/throat, skin/nose and vagina.

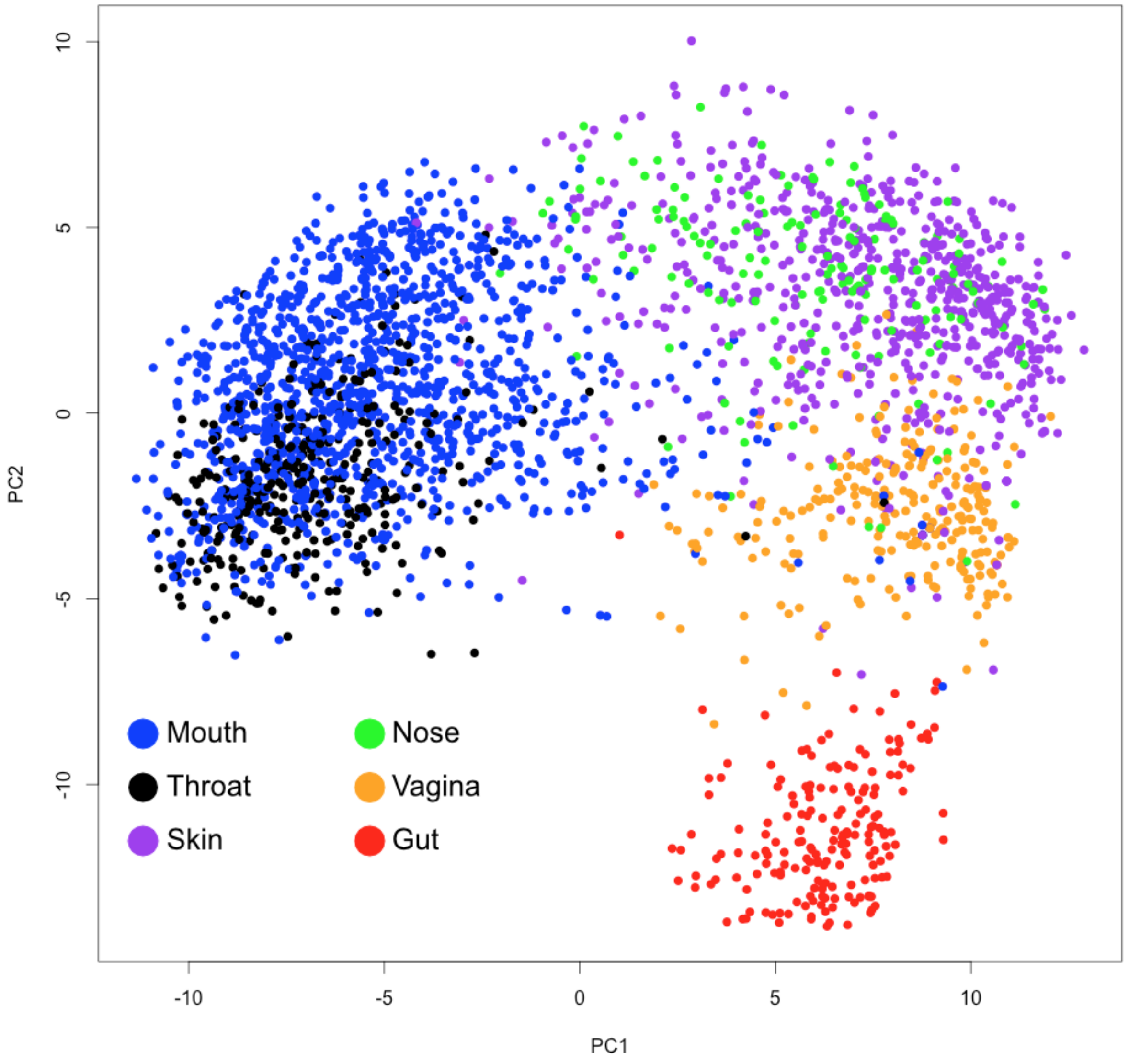

Supplement: Web supplement 9 [file gutjnl-2015-310861-s9.pdf]
